# Predictable, Tunable Protein Production in *Salmonella* for Studying Host-Pathogen Interactions

**DOI:** 10.3389/fcimb.2017.00475

**Published:** 2017-11-16

**Authors:** Kendal G. Cooper, Audrey Chong, Tregei Starr, Ciaran E. Finn, Olivia Steele-Mortimer

**Affiliations:** Laboratory of Bacteriology, Rocky Mountain Laboratories, National Institutes of Allergy and Infectious Diseases, National Institutes of Health, Hamilton, MT, United States

**Keywords:** synthetic biology, *Salmonella*, promoter, plasmid, intracellular, fluorescent protein

## Abstract

Here we describe the use of synthetic genetic elements to improve the predictability and tunability of episomal protein production in *Salmonella*. We used a multi-pronged approach, in which a series of variable-strength synthetic promoters were combined with a synthetic transcriptional terminator, and plasmid copy number variation. This yielded a series of plasmids that drive uniform production of fluorescent and endogenous proteins, over a wide dynamic range. We describe several examples where this system is used to fine-tune constitutive expression in *Salmonella*, providing an efficient means to titrate out toxic effects of protein production.

## Introduction

The synthetic biology approach to the design and fabrication of biological systems is yielding a wealth of synthetic parts that have great potential for use in biomedical research (Weber and Fussenegger, 2012). For example, the use of synthetic promoters to replace their native counterparts in bacteria can dramatically increase the yield of proteins, and this approach can also be used to ensure consistent and predictable levels of proteins for other applications (Mijakovic et al., 2005). In an elegant demonstration of the potential power of rational design, a set of tunable constitutive bacterial promoters were shown to result in highly predictable production of GFP over several orders of magnitude in *Escherichia coli* (*E. coli*; Davis et al., 2011). A key design feature in this series of promoters, is the inclusion of insulating regions to effectively isolate them from the influence of endogenous bacterial regulatory elements. Thus, expression from these promoters is predicted to be highly reproducible even when used in different genetic contexts or environmental conditions.

*Salmonella enterica* serovar Typhimurium (*Salmonella* Typhimurium) is a gut adapted facultative intracellular pathogen that is a common cause of bacterial foodborne disease. Colonization of the intestinal lumen and host cells, is dependent on highly orchestrated metabolic adaption and virulence factor expression. For example, the ability to invade and replicate inside non-phagocytic cells is dependent on the *Salmonella* Pathogenicity Island 1 (SPI1)-regulon that encodes for a Type III Secretion System, T3SS1. Effector proteins translocated into host cells by T3SS1 are responsible for many of the gastrointestinal symptoms of infection, with one such effector, SopB, contributing to intestinal inflammation, invasion and activation of the pro-survival kinase Akt (Galyov et al., 1997; Steele-Mortimer et al., 2000; Zhou et al., 2001). In epithelial cells, *Salmonella* survive and replicate within two distinct niches, either contained by a modified phagosome known as the *Salmonella* containing vacuole (SCV) or free in the cytosol. Vacuolar and cytosolic bacteria are transcriptionally distinct and have different growth kinetics (Knodler et al., 2010; Malik-Kale et al., 2012). Due to the heterogeneous nature of intracellular *Salmonella*, single cell analysis is an important tool for studying this aspect of *Salmonella* pathogenesis. This approach often relies on genetically encoded fluorescent proteins, such as GFP. However, production of these proteins in the bacteria, can have a significant impact on their ability to survive and replicate both *in vivo* and *in vitro* (Lissemore et al., 2000; Wendland and Bumann, 2002; Rang et al., 2003).

GFPmut3 is a bright variant of GFP with a rapid chromophore maturation rate, which has been widely used as a fluorescent reporter in Gram-negative bacteria (Cormack et al., 1996; Iizuka et al., 2011). In *Salmonella* Typhimurium, a commonly used constitutive promoter is the 5′ region of *rpsM* (P*rpsM*), which encodes for the ribosomal protein S13 (Valdivia and Falkow, 1996). However, P*rpsM* activity can be affected by the external environment (e.g., stress; Henard et al., 2014) and cannot readily be adapted to yield a controlled range of strengths (Mijakovic et al., 2005). Plasmid-based expression of GFPmut3 using this relatively strong promoter region can lead to detrimental effects on the ability of *Salmonella* Typhimurium to interact with host cells (Rang et al., 2003; Knodler et al., 2005).

Here, we have constructed a series of tunable expression plasmids using the previously described ProSeries set of promoters (Davis et al., 2011) and combining them with a synthetic transcriptional terminator as well as different plasmid copy number (origins of replication). We show that these plasmids drive uniform protein production over a wide dynamic range, indicating that this tractable expression platform provides an efficient means to identify and utilize the most appropriate promoter strengths for a given application.

## Materials and methods

### Bacterial cultures and growth conditions

*Salmonella enterica* serovar Typhimurium SL1344 and derivatives were used in all experiments (Table 1). Bacteria were grown on LB agar supplemented with streptomycin (100 μg/mL). Plasmids were introduced by electroporation and selection on carbenicillin (50 μg/mL). Strain stocks were frozen in 15% glycerol and stored at −80°C. Fresh plates were streaked from glycerol stocks every week and stored at 4°C except for *hilA* overexpressing strains, which, due to phenotype instability, were not frozen; experiments were done from fresh transformant colonies selected on carbenicillin plates. Overnight cultures were prepared by inoculating one colony into 2 mL LB-Miller broth with selective antibiotics, in a 14-mL polypropylene round-bottom tube (Becton Dickinson) with a loose cap. Cultures were incubated at 37°C in a shaking incubator (225 rpm) for 16–18 h. For sub-culturing (SPI-1 inducing conditions), 0.3 mL of the overnight culture was inoculated into 10 mL LB-Miller broth (no antibiotics unless indicated), in a 125 mL Erlenmeyer flask, and incubated at 37°C with shaking at 225 rpm for 3.5 h or until late log phase. For plate-reader growth assays, a Tecan Infinite 200 Pro plate reader was used. Overnight cultures were diluted 1:25 in fresh LB-Miller and 200 μL were aliquoted in triplicate into 96-well plates and grown with shaking at 37°C.

**Table 1:** Strains and Plasmids used in this study.

**Strain or plasmid**	**Genotype or features**	**References**
**STRAINS**		
SL1344	*hisG46, xyl, rpsL*	Hoiseth and Stocker, 1981
Δ*sopB* (aka Δ*sigDE*)	Δ*sopB* (residues 6–561), IGR^a^, and *sigE* (residues 1–110)	Knodler et al., 2006
Δ*hilA*	Δ*hilA* (KM011)	Main-Hester et al., 2008
**PLASMIDS**		
pFPV25.1	ColE1 *ori*, P*rpsM.gfpmut3* expression	Valdivia and Falkow, 1996
pFPV25.1-TT	ColE1 *ori*, P*rpsM.gfpmut3* expression with synTT	This study
pFPV-*mCherry*	ColE1 *ori*, P*rpsM.mCherry* expression	Drecktrah et al., 2008
pFPV-*mCherry*-TT	ColE1 *ori*, P*rpsM.mCherry* expression with synTT	This study
pWSK29	pSC101 *ori*	Wang and Kushner, 1991
pWSKDE-2xHA	pSC101 *ori*, P*sopB.sopB2xHA-sigE* expression	Knodler et al., 2009
pWSK29ΔP*lac*	pWSK29 without P*lac* promoter	This study
pCON-ProA.*gfp*	ColE1 *ori*, ProA.*gfpmut3* expression	This study
pCON-ProB.*gfp*	ColE1 *ori*, ProB.*gfpmut3* expression	This study
pCON-ProC.*gfp*	ColE1 *ori*, ProC.*gfpmut3* expression	This study
pCON-ProD.*gfp*	ColE1 *ori*, ProD.*gfpmut3* expression	This study
pCON1-ProA.*gfp*	ColE1 *ori*, ProA.*gfpmut3* expression with synTT	This study
pCON1-ProB.*gfp*	ColE1 *ori*, ProB.*gfpmut3* expression with synTT	This study
pCON1-ProC.*gfp*	ColE1 *ori*, ProC.*gfpmut3* expression with synTT	This study
pCON1-ProD.*gfp*	ColE1 *ori*, ProD.*gfpmut3* expression with synTT	This study
pCON-ProA.*mCherry*	ColE1 *ori*, ProA.*mCherry* expression	This study
pCON-ProB.*mCherry*	ColE1 *ori*, ProB.*mCherry* expression	This study
pCON-ProC.*mCherry*	ColE1 *ori*, ProC.*mCherry* expression	This study
pCON-ProD.*mCherry*	ColE1 *ori*, ProD.*mCherry* expression	This study
pCON1-ProA.*mCherry*	ColE1 *ori*, ProA.*mCherry* expression with synTT	This study
pCON1-ProB.*mCherry*	ColE1 *ori*, ProB.*mCherry* expression with synTT	This study
pCON1-ProC.*mCherry*	ColE1 *ori*, ProC.*mCherry* expression with synTT	This study
pCON1-ProD.*mCherry*	ColE1 *ori*, ProD.*mCherry* expression with synTT	This study
pCON-ProA.*sopB*	ColE1 *ori*, ProA.*sopB2xHA-sigE* expression	This study
pCON-ProB.*sopB*	ColE1 *ori*, ProB.*sopB2xHA-sigE* expression	This study
pCON-ProC.*sopB*	ColE1 *ori*, ProC.*sopB2xHA-sigE* expression	This study
pCON-ProD.*sopB*	ColE1 *ori*, ProD.*sopB2xHA-sigE* expression	This study
pCON-ProA.*hilA*	ColE1 *ori*, ProA.*hilA-HA* expression	This study
pCON-ProB.*hilA*	ColE1 *ori*, ProB.*hilA-HA* expression	This study
pCON-ProC.*hilA*	ColE1 *ori*, ProC.*hilA-HA* expression	This study
pCON-ProD.*hilA*	ColE1 *ori*, ProD.*hilA-HA* expression	This study
pCON2-ProA.*hilA*	pSC101 *ori*, ProA.*hilA-HA* expression	This study
pCON2-ProB.*hilA*	pSC101 *ori*, ProB.*hilA-HA* expression	This study
pCON2-ProC.*hilA*	pSC101 *ori*, ProC.*hilA-HA* expression	This study
pCON2-ProD.*hilA*	pSC101 *ori*, ProD.*hilA-HA* expression	This study
pMPMA3ΔPlac-*gfp*	P15A *ori*, promoterless *gfp* plasmid	Finn et al., 2017
pCHAR1	P15A *ori*, P*uhpT*−144 to−1 transcriptional *gfp* reporter	This study
pCHAR1-ProB.*mCherry*	P15A *ori, PuhpT*−144 to−1 transcriptional *gfp* reporter + ProB.*mCherry* with synTT	This study
pCHAR2-ProB.*mCherry*	P15A *ori, PuhpT*−158 to−1 transcriptional *gfp* reporter + ProB.*mCherry* with synTT	This study

a *IGR, intergenic region*.

### Mammalian cell culture

HeLa (human cervical adenocarcinoma, ATCCL-2) cells were grown at 37°C in 5% CO_2_ in complete growth medium: Eagle's minimal essential medium (Mediatech) supplemented with 10% (v/v) heat-inactivated fetal bovine serum (Thermo Fischer Scientific), 2 mM L-glutamine and 1 mM sodium pyruvate. Cells were passaged every 3–4 days and used for experiments within 15 passages of receipt from ATCC.

### Construction of plasmids

The ProSeries promoter sequences were previously described (Davis et al., 2011). The transcriptional terminator (synTT) used was Bba_B0015 of the Registry of Standard Biological Parts (parts.igem.org). All plasmid ligation reactions were carried out using T4 DNA ligase (Promega). Restriction enzymes were obtained from New England Biolabs. The high-fidelity polymerase Phusion was used for all PCR reactions (New England Biolabs). PCR primers were sourced from Integrated DNA technologies. All plasmid constructs were verified by sequencing. Plasmids are listed in Table 1. Oligonucleotide sequences are listed in Table 2.

**Table 2:** Oligonucleotide primers used in this study.

**Primer name**	**Nucleotide sequence (5′ → 3′)**
ProSeries *Xma*I F	NNNNNNATCGATCACAGCTAACACCACGTC
ProSeries *Xba*I R	NNNTCTAGACTAGTACTTTCCTGTGTGACTCTA
*hilA Xba*I F	NNNTCTAGAATGCCACATTTTAATCCTGTTC
*hilA Sph*I HA R	NNNNNNGCATGCTTA*GCCAGAGCCGTAGTCCGGAACGTCGTACGGGTAGCCAGAGCC*CCGTAATTTAATCAAGCG
*hilA Xho*I HA R	NNNNNNCTCGAGTTAGCCAGAGCCGTAGTCC
*sopB Xba*I F	NNNTCTAGAATGCAAATACAGAGCTTCTATCA
*sigE Sph*I R	NNNNNNGCATGCTTATGCATAATGCTCTTTCAATTG
ΔPlac-for	CTTTCCCCGCGGGAAACCTGTCGT
ΔPlac-rev	GGAGCTCCACCGCGGTGGCGG
B0015 *Hind*III F	NNNAAGCTTTATAAACGCAGAAAGGCCC
B0015 *Hind*III R	NNNAAGCTTCCAGGCATCAAATAAAACGAAA
B0015 *Cla*I F	NNNACTGATATAAACGCAGAAAGGCCC
B0015 *Xho*I R	NNNCTCGAGCCAGGCATCAAATAAAACGAAA
ProSeries *Not*I F	NNNNNNGCGGCCGCCACAGCTAACACCACGTC
B0015 *Sac*I R	NNNGAGCTCTATAAACGCAGAAAGGCCC
P*uhpT Not*I F	TAGCTGTGGCGGCCGCAGACCCAGAAGCGTG
P*uhpT* pMPMA3 *Bam*HI R	GGTACCCGGGGATCCGGATTACTCCTGAGCTAATTTTTAT
P*uhpT* pMPMA3 *Not*I F	GGCGGCCGCTCTAGATACACCTCACCTTTTTGC

*Underline, Restriction sites. Italics, GSG-HA-GSG*.

#### pCON-(ProA thru ProD).*gfp*

The ProA, ProB, ProC, and ProD promoters were PCR amplified from the previously described plasmids (Davis et al., 2011), using the oligonucleotides ProSeries *Xma*I F and ProSeries *Xba*I R and cloned into *Xma*I/ *Xba*I digested pFPV25.1 (Valdivia and Falkow, 1996) thus replacing the *rpsM* promoter sequence.

#### pCON1-(ProA thru ProD).*gfp*

synTT was amplified from the from the previously described ProD plasmid (Davis et al., 2011) using oligonucleotides B0015 *Hind*III F and B0015 *Hind*III R and cloned into *Hind*III digested pCON-ProA.*gfp*, pCON-ProB.*gfp*, pCON-ProC.*gfp*, and pCON-ProD.*gfp*.

#### pCON-(ProA thru ProD).*mCherry*

The ProA, ProB, ProC, and ProD promoters, were PCR amplified as above, using the oligonucleotides ProSeries *Xma*I F and ProSeries *Xba*I R and cloned into *Xma*I/*Xba*I digested pFPV*mCherry* (P*rpsM-mCherry*; Drecktrah et al., 2008) to replace the *rpsM* promoter sequence.

#### pCON1-(ProA thru ProD).*mCherry*

synTT was amplified as above using oligonucleotides B0015 *Hind*III F and B0015 *Hind*III R and cloned into *Hind*III digested pCON-ProA.*mCherry*, pCON-ProB*.mCherry*, pCON-ProC*.mCherry*, and pCON-ProD.*mCherry*.

#### pCON-(ProA thru ProD).*sopB*

DNA containing the *sopB-2xHA* and *sigE* ORFs including the intergenic region was amplified using the oligonucleotides *sopB Xba*I 1F and *sigE Sph*I 1R from pWSKDE-2xHA (P*sopB-sopB*; Knodler et al., 2009) and cloned into *Xba*I/*Sph*I digested pCON-ProA.*gfp*, pCON-ProB.*gfp*, pCON-ProC.*gfp*, and pCON-ProD.*gfp*, replacing *gfpmut3* in each construct.

#### pCON-(ProA thru ProD).*hilA*

The *hilA* ORF was PCR amplified from SL1344 genomic DNA with primers which incorporated a c-terminal hemagglutinin (HA) tag, oligonucleotides *hilA Xba*I F and *hilA Sph*I HA R, and cloned into pCON-ProA.*gfp*, pCON-ProB.*gfp*, pCON-ProC.*gfp*, and pCON-ProD.*gfp Xba*I and *Sph*I sites replacing *gfpmut3* in each construct.

#### pCON2-(ProA thru ProD).*hilA*

For low copy *hilA* expression plasmids, pWSK29 (pSC101 *ori*) was used (Wang and Kushner, 1991). First, the P*lac* promoter was removed by inverse PCR with the oligonucleotides ΔP*lac*-for and ΔP*lac*-rev. The resulting amplicon was digested with *Sac*II and self-ligated to give pWSK29ΔPlac. ProA, ProB, ProC, and ProD *hilA-HA* were amplified from pCON-ProA-*hilA*, pCON-ProB-*hilA*, pCON-ProC-*hilA*, and pCON-ProD-*hilA*, respectively, using oligonucleotides *hilA Xba*I F and *hilA Xho*I HA R and cloned into *Xba*I/*Xho*I digested pWSK29ΔPlac.

#### pCHAR1, pCHAR1-ProB.*mCherry*, pCHAR2-ProB.*mCherry*

A truncated *uhpT* promoter region (−144 to −1 bp from start codon) was amplified from SL1344 genomic DNA and cloned into the *Not*I and *Bam*HI sites of the promoterless *gfp* plasmid pMPMA3ΔPlac-*gfp* (Finn et al., 2017). Next, SynTT was amplified as above using oligonucleotides B0015 *Cla*I F and B0015 *Xho*I R and cloned into the *Cla*I and *Xho*I sites, resulting in pCHAR1. ProB-*mCherry*-TT was subcloned from pCON1.ProB.*mCherry* into pCHAR1 using *Not*I and *Sac*I, generating the bidirectional promoter plasmid, pCHAR1-ProB.*mCherry*. The full *uhpT* promoter region (−158 to −1 bp from start codon), containing the full cAMP receptor protein (CAP) binding site (−150 to −130 bp from start codon), was amplified from SL1344 genomic DNA and cloned into the *Not*I and *BamH*I sites of pCHAR1-ProB.*mCherry*, replacing the truncated *uhpT* promoter, to generate pCHAR2-ProB.*mCherry*.

### Promoter activity assay

*Salmonella* were aliquoted into 96-well plates and incubated in the platereader with shaking. OD600, GFP fluorescence (excitation 478 ± 10 nm, emission 515 ± 20 nm) or mCherry fluorescence (excitation 555 ± 10 nm, emission 625 ± 20 nm) were measured every 15 min. For each sample, the promoter activity was calculated as described previously (Kelly et al., 2009). Briefly, the change in fluorescence between two readings during mid log phase of growth (1.5 and 2.5 h) was divided by the average OD600. This measure of promoter activity (per cell synthesis rate) was then normalized to the synthesis rate of the weakest promoter, ProA, resulting in RPU_A_-relative promoter units.

### P*uhpT* induction by exogenous G6P

*Salmonella* carrying pCHAR1-ProB.*mCherry* or pMPMA3ΔPlac-*gfp* were grown overnight and sub-cultured in flasks as described. Glucose-6-phosphate (G6P) (Sigma) was added to late log phase subcultures (3.5 h), aliquoted in triplicate into a 96-well plate, and incubated with agitation at 37°C in the plate reader. OD600, GFP, and mCherry fluorescence were read as described above every 10 min.

### Immunoblotting

Primary antibodies used were mouse monoclonal anti-GFP (11E5, Molecular Probes), mouse monoclonal anti-mCherry (Clontech), mouse monoclonal anti-DnaK (8E2/2, Enzo Life Sciences), mouse monoclonal anti-HA (16B12, Covance), rabbit anti-panAkt (11E7, Cell Signaling), and rabbit anti-phospho Akt Ser473 (D9E, Cell Signaling). Secondary antibodies used were horseradish peroxidase (HRP)-conjugated goat anti-mouse or goat anti-rabbit (Cell Signaling). For chemiluminescent detection, the SuperSignal West Femto Substrate Kit was used according to the manufacturer's instructions (Thermo Fisher Scientific). Images were captured using a Carestream 4000M Pro Image Station and densitometry analysis was performed using ImageJ Software (W.S. Rasband, NIH, version 1.51).

### Flow cytometry

Bacteria were washed once by centrifugation at 8,000 × g for 2 min, resuspended in HBSS and 10–20 μL bacteria were fixed in 500 μL 2.5% (w/v) paraformaldehyde at room temperature for 15 min, centrifuged and finally washed once in PBS. Bacteria were then stained with 10 μM Syto41 (Life Technologies) in PBS for 30 min at room temperature, washed once with PBS by centrifugation, and resuspended in 1 mL PBS for analysis on a BD LSR II flow cytometer (BD Bioscience). Data were analyzed using FlowJo software (Tree Star). Samples were gated on Syto41^+^ events and the % and mean intensity of GFP^+^ or mCherry^+^ events was measured.

### Gentamicin protection assay

These assays were done as described previously (Finn et al., 2017). Briefly, HeLa cells were seeded 20–24 h prior to infection in 24-well plates at 4.5 × 10^4^ cells per well. SPI1-induced *Salmonella* were collected by centrifugation at 8,000 × *g* for 2 min, washed and resuspended in Hank's buffered saline solution (HBSS) and used immediately to infect epithelial cells for 10 min at 37°C at an MOI of ~50. Inoculum CFU counts were checked by plating on LB agar plates. Extracellular bacteria were removed by washing with HBSS and cells were incubated in antibiotic-free complete growth media until 30 min post-infection (pi). Cells were then incubated for 1 h in complete growth media supplemented with L-Histidine (500 μg/mL) and gentamicin (50 μg/mL), followed by complete growth media supplemented with L-Histidine (500 μg/mL) and gentamicin (10 μg/mL) for the remainder of the infection. At indicated time-points, monolayers were lysed in 1 mL of 0.2% (w/v) sodium deoxycholate (DOC) in PBS and viable intracellular bacteria were enumerated by plating on LB agar.

### Akt activation assay

HeLa cells were seeded in 6-well tissue culture plates at a density of 1.5 × 10^5^ cells/well 18–20 h pre-infection. On the day of infection, cells were serum starved for 3 h pre-infection and maintained in serum-free media throughout the assay. Cells were infected as described above. At 60 min pi, cells were washed once in ice-cold HBSS and lysed in ice-cold RIPA buffer (Sigma). Cell lysates were centrifuged at 4°C for 20 min at 16,000 × *g* to pellet cellular debris and the supernatant was transferred to a clean, pre-chilled tube. Total protein concentration was determined using the DC protein assay (BioRad) to ensure equal loading of samples for SDS-PAGE and immunoblotting.

### Immunofluorescence microscopy and image analysis

For experiments in Figure **3C**, HeLa cells were plated 20–24 h prior to infection on glass coverslips in 24-well plates (5.5 × 10^4^ cells per well) and infected as described above. At indicated time points pi, cells were fixed in 2.5% paraformaldehyde (w/v) in PBS for 10 min at 37°C. Cells were permeabilized and blocked in 0.1% (w/v) saponin plus 10% (v/v) normal goat serum in PBS (PBS-SS) for 30 min. Primary and secondary antibodies were rabbit anti-*Salmonella* LPS (1:300; Difco) and AlexaFluor 568-conjugated goat anti-rabbit IgG (1:500; Molecular Probes) diluted in PBS-SS. Coverslips were mounted on glass slides using AntiFade Gold + DAPI (Molecular Probes). Images were captured with the same gain and exposure for each sample on a Photometrics CoolSnap HQ camera using a 60 × /1.4N objective on a Nikon Ti epifluorescence widefield microscope. Post-acquisition analysis of fluorescence intensities was done using the ImageJ software Cell Counter Plugin. Bacteria were selected in the mCherry channel, blind to the GFP content, followed by pixel intensity measurement in the GFP channel. The average background intensity for each field was subtracted from the GFP pixel intensity for each bacterium.

### Differential permeabilization assay and image analysis

HeLa cells were infected as described above. The differential permeabilization assay was performed as previously described (Finn et al., 2017). Briefly, infected HeLa cells were washed three times with KHM buffer (110 mM potassium acetate, 20 mM HEPES, 2 mM MgCl_2_, pH 7.3), and the plasma membrane selectively permeabilized by incubation with 40 μg/mL digitonin (Sigma) in KHM buffer for 1 min at RT, followed by three washes with KHM buffer. Cells were then incubated for 12 min at RT with rabbit anti-Calnexin (Stressgen), to label the cytosolic face of the endoplasmic reticulum in permeabilized cells, and Pacific Blue-conjugated goat anti-*Salmonella* CSA1 antibodies (KPL), to detect cytosolic bacteria. After paraformaldehyde fixation, rabbit anti-Calnexin antibodies were detected with Alexa Fluor 647-conjugated anti-rabbit antibodies. Coverslips were then washed sequentially with PBS and distilled water, and mounted on glass slides in a MOWIOL solution supplemented with 2.5% (w/v) DABCO. Images were acquired with a Carl Zeiss LSM 710 confocal laser-scanning microscope equipped with a Plan APOCHROMAT 63X/1.4 N.A. objective, processed into maximum intensity projections using Zen 2012 SP1 software and assembled using Adobe Photoshop CC. Pixel area and fluorescence intensities were quantified using CellProfiler (Carpenter et al., 2006) from maximum intensity projections. The pixel area occupied by intracellular *Salmonella* was determined by mCherry signal. The 488 (GFP) and 455 (DAPI) nm intensities within the area occupied was quantified and the ratio of each fluorescence signal to pixel area was calculated.

### Live cell imaging and quantification

HeLa cells grown on 24-well glass bottom plates (Greiner Bio-One) were infected as described above with either WT or ΔSPI2 *Salmonella* carrying pCHAR2-ProB*.mCherry*. Cells were imaged using a TiE inverted microscope with Perfect Focus System (Nikon) and custom laser launch (Prairie Technologies). Environmental control was maintained with a stage-top incubation system (Pathology Devices). Beginning at 3 hpi, images were collected on an iXon EMCCD camera (Andor) every 15 min until ~22 hpi using a Plan Fluor 40X/0.75NA objective. GFP intensity plotted vs. time for individual cells was used to determine the doubling time of bacteria in the cytosol. The data was analyzed using an exponential growth, nonlinear regression analysis (least squares fit). Vacuolar fold change was calculated as a ratio of the maximum mCherry intensity per cell vs. intensity at 3 hpi. All post-acquisition image analysis was done using ImageJ software, GraphPad Prism version 7.0a, and Adobe Photoshop (CC v2015.1.2 Adobe).

### Statistics

Statistical significance was determined using one-way ANOVA, followed by Tukey's multiple comparisons test, where indicated. A *P*-value of ≤ 0.05 was considered significant.

## Results

### Evaluation of synthetic constitutive promoters and terminators in *Salmonella*

**Figure 1:**
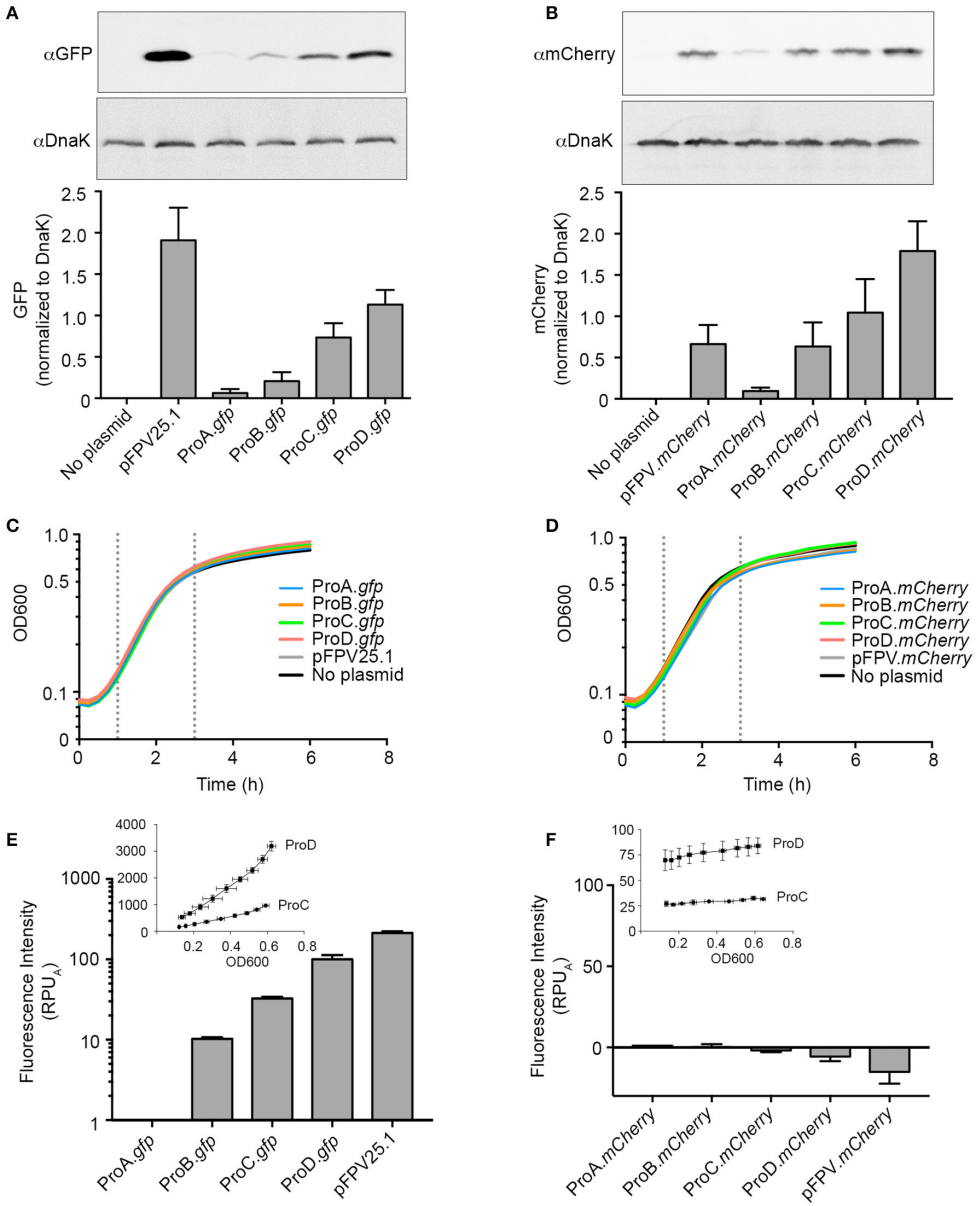
Evaluating synthetic promoter activity in *Salmonella* Typhimurium. **(A,B)** Bacteria harboring the indicated plasmids were grown to late-log phase in LB-Miller broth with aeration. Samples were solubilized and processed for immunoblotting using antibodies to detect GFP **(A)** or mCherry **(B)**. DnaK was used as a loading control. Representative immunoblots are shown (top panels) along with quantification of three experiments by densitometry analysis (bottom panels). Shown is the ratio of GFP or mCherry signal to DnaK signal (mean ± *SD*). **(C,D)** Bacteria were grown in 96-well plates and fluorescence and OD600 measurements were taken every 15 min. Growth curves for strains harboring GFP constructs **(C)** or mCherry constructs **(D)**. Shown is the mean OD600 of three independent experiments. Log phase is observed between 1 h and 3 h post inoculation (dotted lines). **(E,F)**. Relative promoter units normalized to ProA (RPU_A_) were calculated using fluorescence of GFP **(E)** or mCherry **(F)** at 1.5 and 2.5 h time points. The fluorescence intensity for pCON-ProC.*gfp* and pCON-ProD.*gfp* or pCON-ProC.*mCherry* and pCON-ProD.*mCherry* during log phase is plotted against OD600 in the insets (Mean ± *SD, n* = 3).

As a first step in developing a rationally designed expression system for use in *Salmonella* Typhimurium, we selected two unrelated fluorescent proteins, GFPmut3 and mCherry as reporters (Shaner et al., 2004). Both of these are widely used in the *Salmonella* field, often expressed constitutively under P*rpsM* in the same plasmid backbone (pFPV25.1 and pFPV.*mCherry*, respectively; Valdivia and Falkow, 1996; Drecktrah et al., 2008). We began by replacing P*rpsM* in pFPV25.1 with the synthetic promoters ProA, ProB, ProC, or ProD resulting in the plasmids pCON-ProA.*gfp*, pCON-ProB.*gfp*, pCON-ProC.*gfp*, and pCON-ProD.*gfp*, respectively. We also made the analogous series of mCherry plasmids pCON-ProA.*mCherry*, pCON-ProB.*mCherry*, pCON-ProC.*mCherry* and pCON-ProD.*mCherry*. Immunoblotting of lysates of late log phase bacteria harboring the pCON plasmids revealed that the levels of both GFP (Figure 1A) and mCherry (Figure 1B), correlated well with promoter strength, which increases from ProA to ProD (Davis et al., 2011).

Growth curves revealed that constitutive expression of either GFP (Figure 1C) or mCherry (Figure 1D) by any of these plasmids had no detectable effect on cell growth compared to *Salmonella* containing no plasmid. Under these conditions, log phase growth was observed from 1 to 3 h post-inoculation for all strains with a doubling time of 45–55 min. This is somewhat longer than we have previously reported (Ibarra et al., 2010), presumably due to the use here of a reduced-volume 96-well plate format to facilitate measurement of both fluorescence and OD600 for multiple strains over time. The fluorescence measurements allowed us to quantitatively evaluate promoter activity. The protein synthesis rate for each promoter was calculated by dividing the increase in fluorescence intensity between two time points in log phase (1.5 and 2.5 h) by the average OD600 (Kelly et al., 2009; Davis et al., 2011). To allow for better comparison between the different promoters the synthesis rate was normalized to that of ProA, yielding relative promoter units (RPU_A_). In agreement with the published observations in *E. coli* (Davis et al., 2011), the range of synthetic promoter activity spanned two orders of magnitude when GFP was used as a reporter (Figure 1E). However, we were unable to distinguish a difference in promoter activity using mCherry as a reporter (Figure 1F). The reason for this is apparent when fluorescence intensity is plotted against OD600, which reveals that, unlike GFP (Figure 1E, Inset), mCherry fluorescence did not increase with bacterial growth during log phase (Figure 1F, Inset).

**Figure 2:**
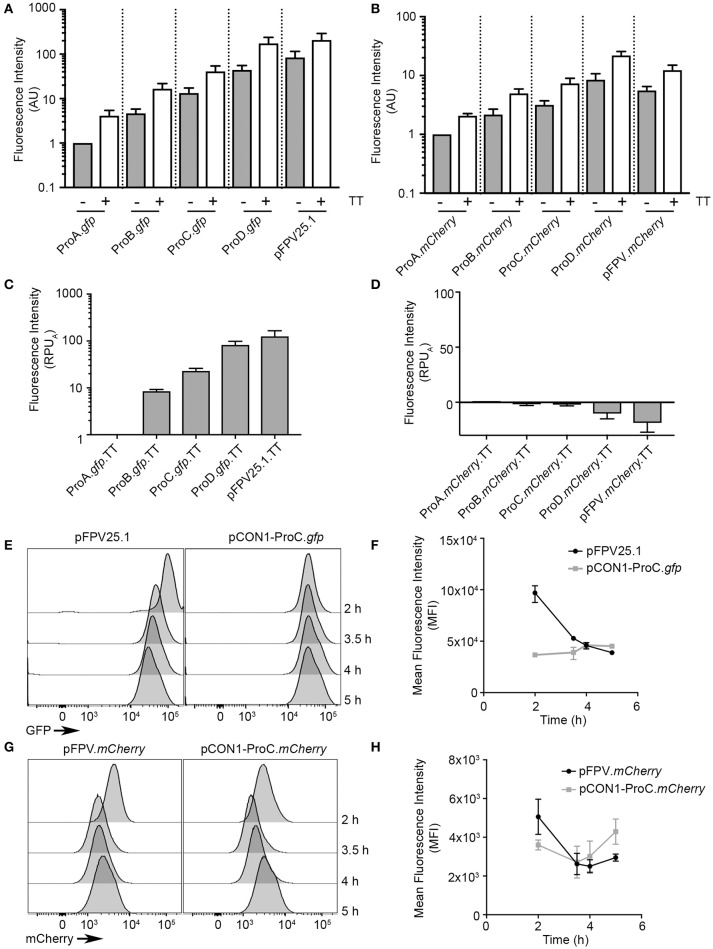
Optimization of fluorescent protein production using a synthetic transcriptional terminator. Bacteria containing constructs with and without a synthetic transcriptional terminator (TT) were grown in 96-well plates and GFP fluorescence **(A)** or mCherry fluorescence **(B)** was measured at 2.5 h of growth (Mean ± *SD, n* = 3). AU, arbitrary units **(C,D)** as in Figure 1 relative promoter units were obtained by normalizing to ProA (RPU_A_) for GFP **(C)** or mCherry **(D)** at 1.5 and 2.5 h time points. **(E–H)** Flow cytometry analysis of bacteria harboring the indicated plasmids. Bacteria were harvested at early log (2 h), late log (3.5 h), early stationary (4 h), and late stationary (5 h) phases. Shown are representative histograms at the indicated time points for GFP constructs **(E)** or mCherry constructs **(G)**. Fluorescence intensities of GFP **(F)** and mCherry **(H)** were plotted at each time point (Mean ± *SD, n* = 3).

The second step in developing these optimized expression vectors was the addition of a synthetic transcriptional terminator (synTT) to each of the plasmids, resulting in the pCON1 series of plasmids: pCON1-ProA.*gfp*, pCON1-ProB.*gfp*, pCON1-ProC.*gfp*, pCON1-ProD.*gfp*, and pFPV25.1-TT along the analogous mCherry plasmids. The addition of synTT resulted in a three- to four-fold increase in fluorescence for bacteria bearing the GFP plasmids (Figure 2A) and a 2- to 2.5-fold increase for those bearing the mCherry plasmids (Figure 2B), at 2.5 h post inoculation. Although addition of the terminator resulted in higher protein production, promoter activity (RPU_A_) was not increased (Figures 2C,D).

**Figure 3:**
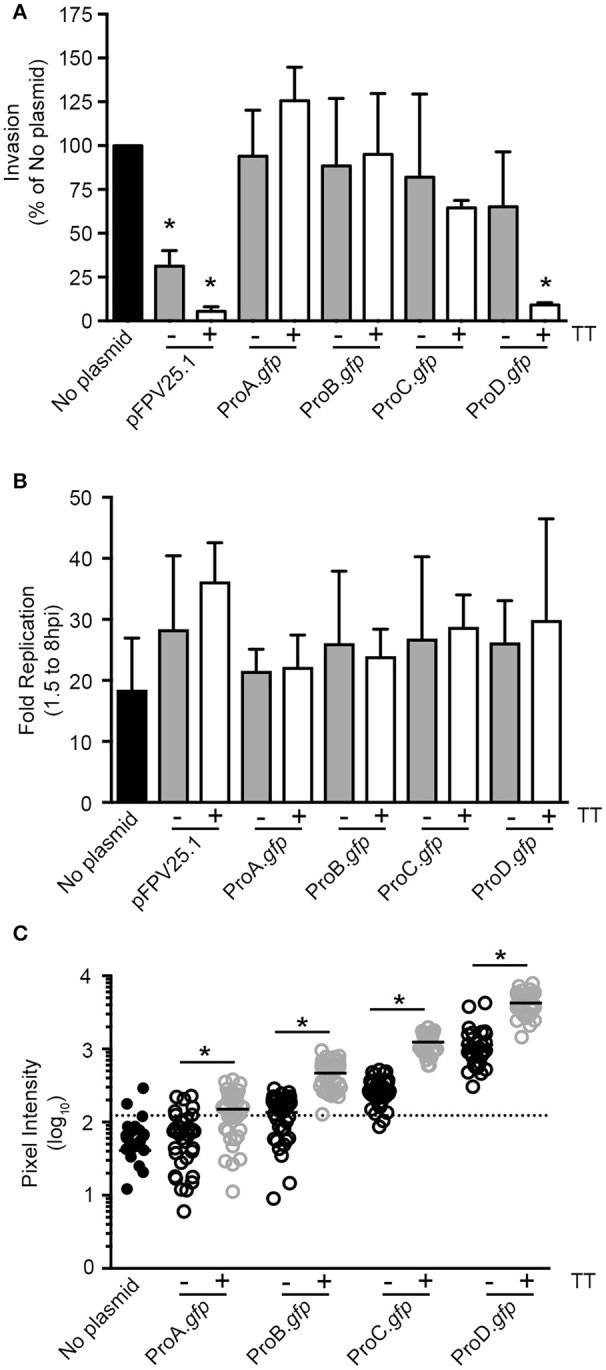
Titration of GFP production in *Salmonella* can eliminate plasmid associated invasion defects. **(A)** Invasion assay in HeLa cells. Values were normalized to bacteria containing no plasmid (Mean ± *SD, n* = 3). **(B)** Replication assay in HeLa cells (Mean ± *SD, n* = 3). **(C)** GFP fluorescent intensities of intracellular bacteria at 1.5 hpi. Shown is data from one representative experiment; each dot represents one bacterial cell. The means are indicated. The threshold was set at three-fold the mean intensity of bacteria with no plasmid (dotted line). ^*^Signifies *P*-value ≤ 0.05.

The above experiments show on a population level, how the synthetic promoters and terminator affect protein production, however, they provide no information on cell-to-cell variability. Therefore, to evaluate heterogeneity at the single cell level we used flow cytometry to compare the fluorescence intensity of individual bacteria over time. We selected the pCON1-ProC.*gfp* and pCON1-ProC.*mCherry* plasmids for single cell analysis, since the ProC promoter resulted in GFP fluorescence levels that were readily detectable by fluorescence microscopy without negatively impacting invasion (Figure 3A). Bacteria were harvested and fixed at 2, 3.5, 4, and 5 h post-inoculation, which under these growth conditions correspond to log phase, late-log phase, early stationary phase and stationary phase, respectively. The mean fluorescence intensity of *Salmonella* harboring pCON1-ProC.*gfp* was similar at all four time points (range from 36,601 ± 233 to 46,013 ± 2465, mean ± *SD*), whereas bacteria harboring pFPV25.1 were significantly brighter at 2 h (96,994 ± 8,395) than later time points (Figures 2E,F). In contrast, bacteria harboring the mCherry constructs, pFPV*.mCherry* or pCON1-ProC.*mCherry* fluctuated between each of the time points (Figures 2G,H). Thus, by both population based and single cell based analysis, the synthetic *gfp* expression construct produced the most consistent, predictable fluorescent protein production.

### Titration of GFP production in *Salmonella* can eliminate plasmid-associated invasion defects

Given the wide range of GFP production levels obtained using synthetic elements, we next assessed which *gfp* constructs are compatible for infection studies using the well-established HeLa cell invasion model. As previously shown, strains harboring pFPV25.1 had a significant invasion defect (Knodler et al., 2005) and this was exaggerated by the addition of the synTT (Figure 3A), which increased GFP production (Figure 2A). For synthetic promoter driven GFP production, only the strain harboring pCON1-ProD.*gfp*, which together with pFPV25.1-TT produces the largest amounts of GFP (Figure 2A), had an invasion defect. All others, despite profound differences in GFP production spanning over two orders of magnitude, had no detectable effect on invasion (Figure 3A). Furthermore, we observed no impact on intracellular replication in HeLa cells (Figure 3B).

Altogether, the preceding experiments showed that *Salmonella* bearing the pCON expression vectors produced GFP over a wide spectrum of levels, without any effect on growth or invasion. Since our goal was to use these plasmids to study intracellular *Salmonella*, we also evaluated intracellular bacteria by fluorescence microscopy (Figure 3C and Table 3). The mean fluorescence intensity (MFI) of *Salmonella* producing GFP under the control of the weakest synthetic promoter, ProA, was slightly higher than that of bacteria bearing no plasmid (78 ± 25 vs. 40 ± 1, mean ± *SD*). Thereafter, the MFI increased with promoter strength (ProA < ProB < ProC < ProD) and was further increased, approximately three-fold, for each promoter in the presence of the synTT. The highest MFI (3,706 ± 1,750), was observed in bacteria bearing pCON1-ProD.*gfp*. Using a threshold of “3X the MFI of bacteria bearing no plasmid,” >90% of *Salmonella* bearing pCON1-ProB.*gfp*, pCON-ProC.*gfp*, pCON1-ProC.*gfp*, pCON-ProD.*gfp*, and pCON1-ProD.*gfp* plasmids were GFP positive (Table 3). Thus, GFP intensity correlated well with synthetic promoter strength and was further increased in the presence of the synthetic transcriptional terminator.

**Table 3:** GFP fluorescence analysis of single intracellular bacteria.

	**MFI (Mean ± *SD*)**	**% GFP positive ± *SD***
No plasmid	40 ± 1	10 ± 5
pCON-ProA.*gfp*	78 ± 25	25 ± 13
pCON1-ProA.*gfp*	183 ± 113	60 ± 25
pCON-ProB.*gfp*	152 ± 77	59 ± 30
pCON1-ProB.*gfp*	525 ± 220	98 ± 2
pCON-ProC.*gfp*	305 ± 130	93 ± 8
pCON1-ProC.*gfp*	1,113 ± 405	97 ± 2
pCON-ProD.*gfp*	890 ± 416	98 ± 2
pCON1-ProD.*gfp*	3,706 ± 1,750	100 ± 0

*Threshold is defined as 3X the MFI of bacteria with no plasmid*.

### Tunable constitutive expression of the *Salmonella* effector SopB

**Figure 4:**
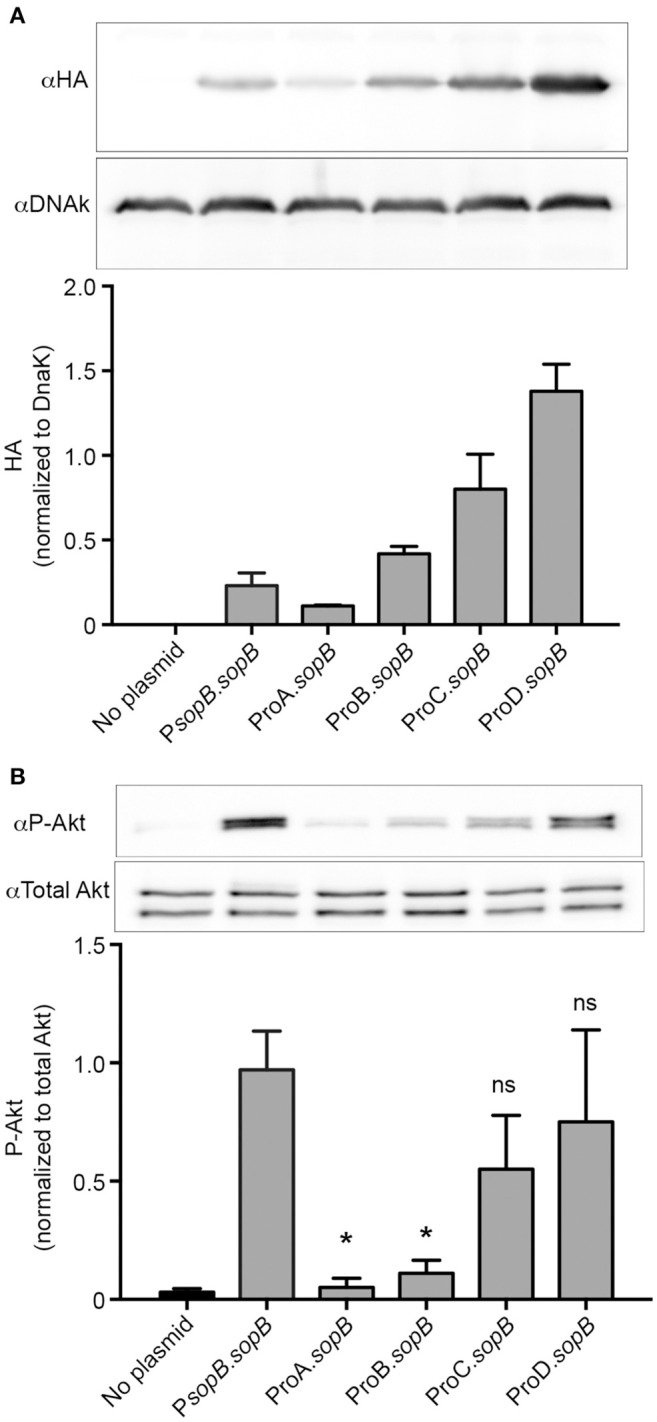
Tunable constitutive expression of the T3SS1 effector, SopB. **(A)** Δ*sopB* bacteria harboring the indicated plasmids were grown to late-log phase in LB-Miller broth with aeration. Samples were solubilized and processed for immunoblotting using antibodies to detect HA and DnaK. Representative immunoblots (top panel) are shown along with quantification of three experiments by densitometry analysis (bottom panel). Shown is the ratio of HA signal to DnaK signal (mean ± *SD*). **(B)** Infected HeLa cells were solubilized at 60 min pi and processed for immunoblotting using antibodies to detect phospho-Akt and total Akt. Representative immunoblots (top panel) are shown along with quantification of three experiments by densitometry analysis (bottom panel). Shown is the ratio of phospho-Akt signal to total Akt signal (mean ± *SD*). ^*^Signifies *P*-value ≤ 0.05.

Thus far we have shown that the pCON plasmid series enables tunable, predictable and homogeneous production of GFP. Next, we wanted to take advantage of the pCON-based tunable expression system as a way to study *Salmonella* effector protein function. We selected SopB for these experiments, because, upon delivery into the host cell by T3SS1, the activity of this effector protein can be readily quantified by phospho-specific immunoblotting for phosphorylated AKT (Steele-Mortimer et al., 2000). To express SopB together with its cognate chaperone, SigE, we made constructs with the *sopB-sigE* operon under the control of each of the ProSeries promoters, and including a SopB C-terminal 2xHA tag, yielding pCON-ProA.*sopB*, pCON-ProB.*sopB*, pCON-ProC.*sopB*, and pCON-ProD.*sopB*. These plasmids were introduced into a SopB-SigE deletion mutant (Δ*sopB*; Knodler et al., 2006). To evaluate the levels of SopB-2xHA protein, lysates were prepared from SPI1-induced bacteria. Under these conditions SopB expressed under the control of its native promoter from a low copy number plasmid (pWSKDE-2xHA or P*sopB*.*sopB*) functionally complements Δ*sopB* (Knodler et al., 2009). Immunoblotting revealed that, production of HA-tagged SopB under the control of the ProSeries promoters was consistent with promoter strength, with ProA.*sopB* producing the lowest levels and ProD.*sopB* producing the highest levels (Figure 4A). Analysis of SopB levels produced by the pCON plasmids revealed that at late log phase (as used for invasion), the amounts of SopB produced by ProB.*sopB* were most similar to the amount produced by the native promoter (P*sopB.sopB*).

We next compared the ability of constitutively vs. natively expressed SopB to functionally complement the Δ*sopB* mutant. In HeLa cells, SopB-dependent Akt phosphorylation peaks at ~1 hpi (Steele-Mortimer et al., 2000; Cooper et al., 2011). When the Δ*sopB* mutant is complemented with plasmid borne SopB expressed under its native promoter (P*sopB*.*sopB*), Akt phosphorylation is restored (Figure 4B). Notably, ProB driven production of SopB, while producing similar levels of the effector to the native promoter, did not restore Akt phosphorylation (Compare Figures 4A,B). However, constitutively expressed SopB produced by either ProC or ProD restored the ability to induce Akt phosphorylation. Thus, the variable strength ProSeries of constitutive promoters can facilitate the identification of optimal constitutive expression levels needed to complement SopB function. Further fine-tuning, if necessary could be achieved by the incorporation of the synthetic TT as demonstrated for GFP and mCherry.

### Tunable constitutive expression of the SPI1 master regulator *hilA*

**Figure 5:**
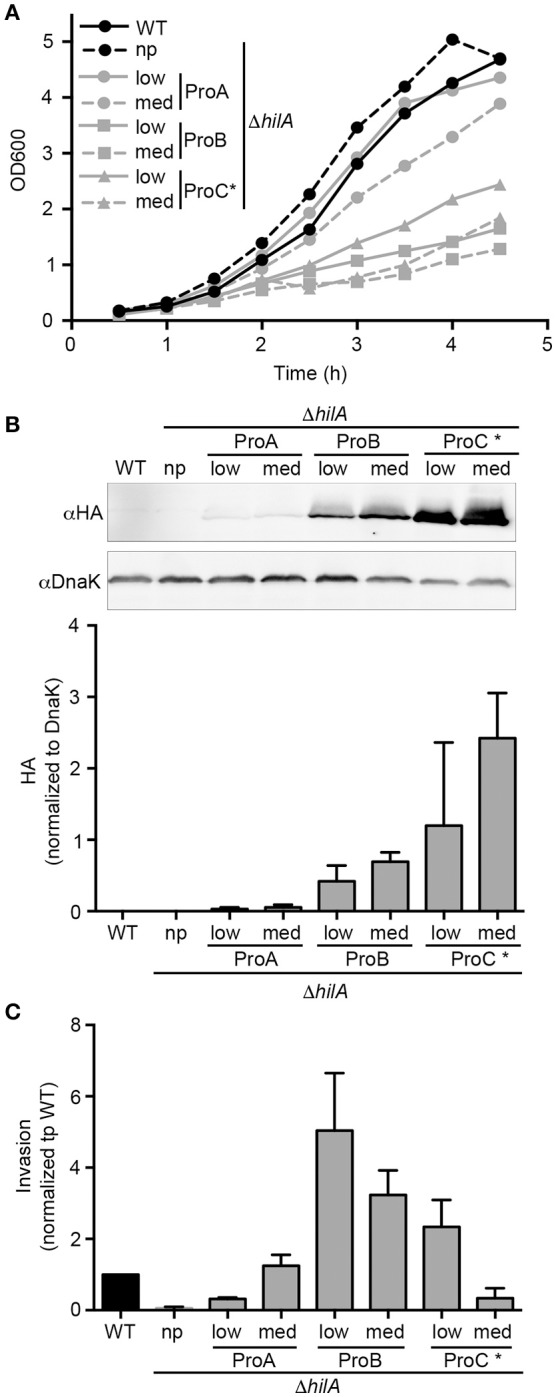
Tunable constitutive expression of the T3SS1 regulator, HilA. **(A)** Bacteria were grown in 125 mL flasks and OD600 measurements were taken every 30 min. Growth curves for bacteria harboring the indicated plasmids. Shown is mean OD600 of three independent experiments. **(B)** Late log phase samples were solubilized and processed for immunoblotting using antibodies to detect HA and DnaK. Representative immunoblots are shown (top panels) along with quantification of three experiments by densitometry analysis (bottom panels). Shown is the ratio of HA signal to DnaK signal (mean ± *SD, n* = 3). WT *Salmonella* (the chromosomal *hilA* is not HA-tagged) were used as a negative control for HA detection. **(C)** Invasion assay in HeLa cells. Values were normalized to WT (Mean ± *SD, n* = 3). np, no plasmid; low, low copy plasmid; med, medium copy plasmid. ^*^Expression of *hilA* under ProC resulted in inconsistent growth and protein levels between experiments.

Constitutive expression of transcriptional regulators, such as those controlling *Salmonella* virulence genes, can be used to identify pathways affected by these regulators as well as to interrogate their roles in transcriptional cross-talk. HilA is a key regulator of the SPI1-regulon, and ultimately controls expression of genes encoding the T3SS1 secretion apparatus and secreted effectors (Bajaj et al., 1995, 1996). The level of SPI1 gene expression is dependent on the level of HilA, and high levels of expression of *hilA* have a fitness cost to the bacteria through increased production of T3SS1 (Sturm et al., 2011). Thus, this regulator is a good candidate for optimization of expression with a tunable promoter pCON plasmid series. Since we expected small changes in expression levels of *hilA* to have significant impacts on fitness, we introduced an additional level of tuning by using two plasmid backbones, one medium copy (ColE1 *ori*, ≈ 15–20 copies/cell) and one low copy (pSC101 *ori*, ≈ 5 copies/cell). The resultant plasmids were: pCON-(ProA thru ProD).*hilA* (medium copy number) and pCON2-(ProA thru ProD).*hilA* (low copy number). The plasmids were introduced into a Δ*hilA* background, to allow analysis of the impact of constitutive *hilA* expression. Immediately after transformation, we observed a growth defect in all transformants except those producing HilA under the control of ProA (Table 4). Expression of *hilA* under the control of the strongest promoter, ProD, resulted in punctiform colonies that grew poorly in liquid cultures. Due to these profound growth defects, the ProD constructs were excluded from further analyses. As shown in Figure 5A, a Δ*hilA* mutant replicates faster than WT *Salmonella* in broth cultures due to the lack of T3SS1 production (Sturm et al., 2011). Constitutive expression of *hilA* in the Δ*hilA* strain under the control of the weakest promoter (ProA) recapitulated the WT growth rate when the low copy number backbone was used. When constitutive expression of *hilA* was increased, either by using the stronger promoter ProB or by increasing the plasmid copy number, growth retardation was exacerbated. However, when *hilA* expression level was further increased using the ProC promoter the growth phenotype was inconsistent.

**Table 4:** Colony size of transformants.

	**ProA-*hilA***	**ProB-*hilA***	**ProC-*hilA***	**ProD-*hilA***
pCON (medium copy)	+++	++	+	+/–
pCON2 (low copy)	+++	++	+	+/–

*+, smaller than ++; ++, smaller than +++; +++, normal size (comparable to WT); +/−, punctiform*.

Overexpression of *hilA* under the control of its native promoter causes hyper-invasiveness into host cells (Bajaj et al., 1995; Sturm et al., 2011). To examine whether constitutive expression of *hilA* can recapitulate this effect, HeLa cells were infected with strains harboring the *hilA* plasmid series, and invasion was measured using the gentamicin protection assay. Immunoblotting of bacterial lysates, prepared from bacteria grown under SPI1-inducing conditions (as used for invasion), revealed that HilA protein levels reflected a combination of both promoter strength and plasmid copy number (Figure 5B). Although, it should be noted that, expression under the control of the strongest promoter, ProC, resulted in variable levels of HilA. In line with published work, our invasion assay confirmed that the Δ*hilA* strain is non-invasive (Figure 5C). Complementation of this strain with constitutive expression of *hilA* from the plasmids pCON2-ProB.*hilA*, pCON-ProB.*hilA* or pCON2-ProC.*hilA* lead to increased invasion by up to ≈ 5-fold compared to the WT strain. Further increase in HilA production above this level, by pCON-ProC.*hilA*, resulted in an invasion defect compared to WT, thus revealing a threshold of *hilA* expression above which invasion is compromised (Figure 5C). Thus, by combining the ProSeries of promoters with different copy number plasmids, we were able to fine-tune expression levels of *hilA* to find the optimum conditions for constitutive expression.

### Design of a bidirectional environmental sensor for single cell analysis

Finally, we wished to apply the synthetic elements approach to developing a bidirectional environmental sensor for use in *Salmonella*. Previously we have described a fluorescent reporter, P*uhpT-gfp*, that is specifically induced in the cytosolic subpopulation of *Salmonella* in epithelial cells (Finn et al., 2017). This GFP based cytosolic hexose phosphate activated reporter (CHAR) is induced by the presence of glucose-6-phosphate (G6P), which is found in the cytosol but not the SCV (Finn et al., 2017). While this single-color reporter enables detection of the cytosolic population, visualization of the total bacterial population requires a constitutively produced second fluorophore, which could have a negative impact on bacterial fitness.

**Figure 6:**
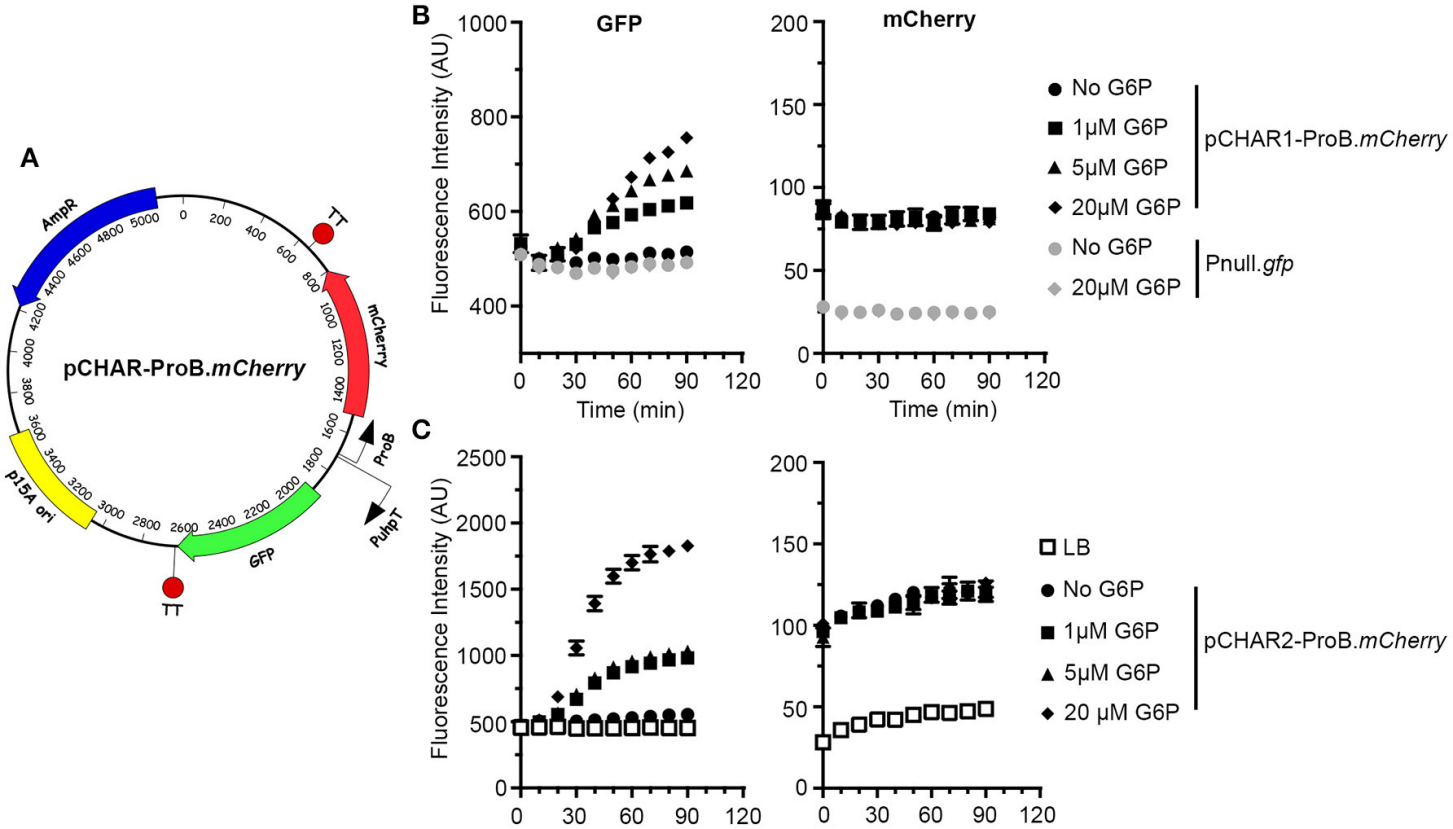
Design of the bidirectional environmental sensor plasmids. **(A)** Plasmid map of pCHAR-ProB.*mCherry* constructs. mCherry expression is driven by ProB; GFP expression is controlled by the glucose-6-phosphate (G6P) responsive promoter, P*uhpT*. **(B,C)** Induction of GFP fluorescence is G6P dependent. Late-log phase bacteria harboring pCHAR1-ProB.*mCherry* or Pnull-*gfp* **(B)** or pCHAR2-ProB.*mCherry* **(C)** were treated with 0–20 μM G6P and GFP (left) and mCherry (right) fluorescence was monitored in a plate-reader at 10 min intervals. Shown is data representative of three independent experiments with mean ± *SD* from triplicate samples.

To address the need for a two-color cytosolic reporter we designed two bidirectional vectors with constitutive mCherry production under the control of the synthetic ProB promoter and GFP production under the control of P*uhpT* (Figure 6A). SynTT was included at the 3′ end of both fluorescent genes. To add another level of tunability to this system we used both full-length and truncated (lacking 14 bp at the 5′ end) versions of the *uhpT* promoter resulting in pCHAR2-ProB-*mCherry* and pCHAR1-ProB.mCherry respectively. In broth grown cultures, GFP fluorescence in bacteria harboring either of these reporter plasmids responded in a dose dependent manner to exogenous G6P, while mCherry fluorescence was unchanged (Figures 6B,C). For a “no fluorescence control” we included bacteria bearing the promoterless pMPMA3ΔPlac-*gfp* (Pnull.*gfp*). Comparison of the maximal fluorescence intensity of GFP following induction with G6P revealed that bacteria harboring pCHAR2-ProB.*mCherry* were ~2.5-fold brighter than those harboring pCHAR1-ProB.*mCherry* (1,827 ± 44 vs. 689 ± 112), confirming that the full length *uhpT* promoter is stronger.

**Figure 7:**
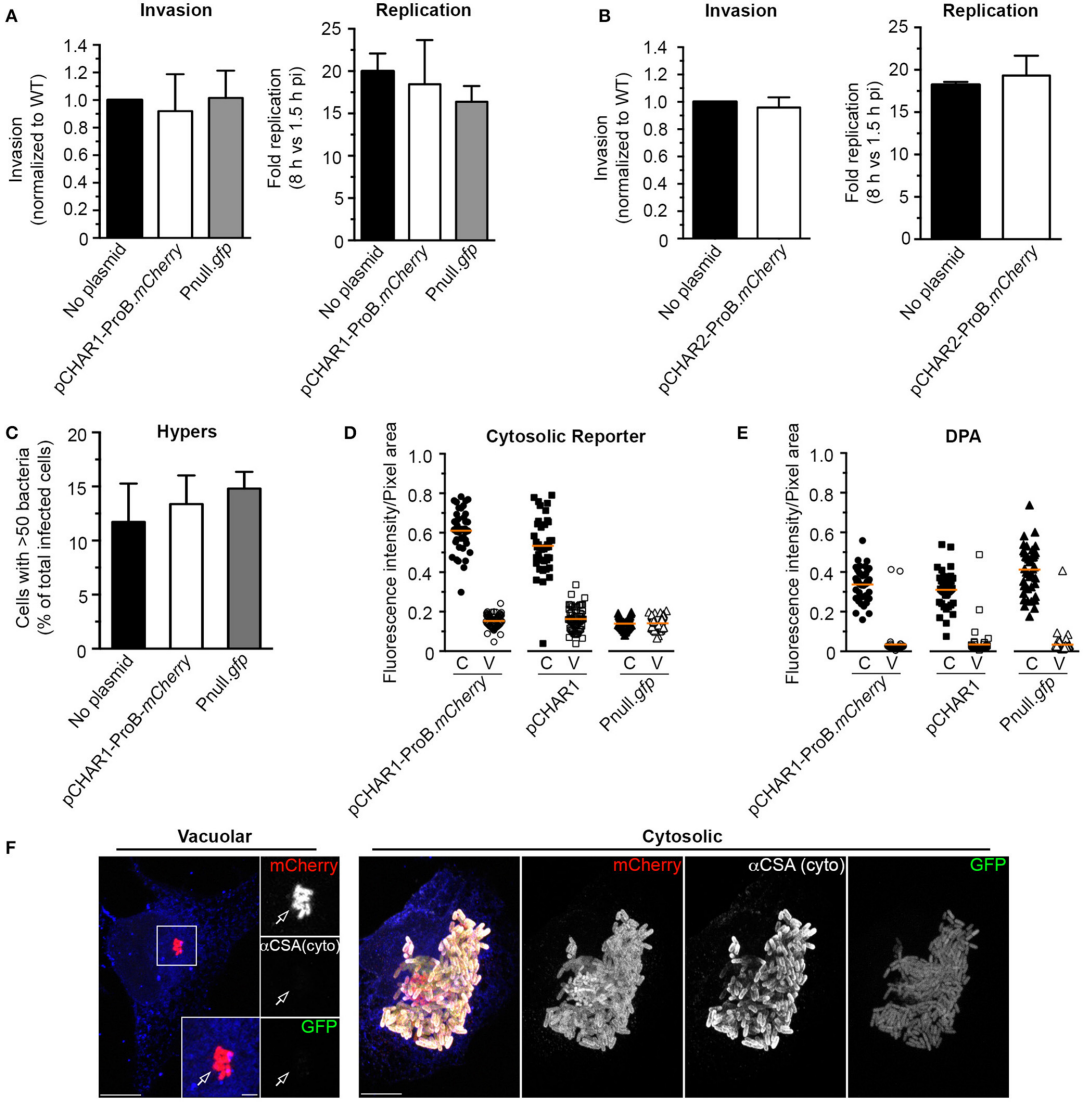
The bidirectional sensor plasmid pCHAR1-ProB.*mCherry* is a reporter for cytosolic *Salmonella*. **(A,B)** Invasion and replication (1.5–8 hpi) assays in HeLa cells of bacteria harboring either pCHAR1-ProB.*mCherry* or Pnull.*gfp* **(A)**, or pCHAR2-ProB.*mCherry* **(B)**. Values were normalized to bacteria containing no plasmid (mean ± *SD, n* = 3). **(C)** Percentage of infected cells containing hyper-replicating *Salmonella* bearing either pCHAR1-ProB.*mCherry* or Pnull.*gfp* at 6 hpi. Infected cells containing >50 bacteria/cell were scored (Mean ± *SD, n* = 3). **(D,E)** Fluorescence intensities in HeLa cells of vacuolar or cytosolic hyper-replicating *Salmonella*. Cells infected with bacteria harboring pCHAR1-ProB.*mCherry*, pCHAR1 or Pnull.*gfp* were fixed at 6 hpi and cytosolic bacteria were identified either by GFP signal **(D)** or by digitonin permeabilization (DPA) followed by antibody labeling **(E)**. GFP fluorescence intensities were measured within pixel areas identified using either mCherry signal (pCHAR1-ProB.*mCherry*) or from fluorescent immunolabeling (pCHAR1 and Pnull.*gfp*). Each data point represents an infected cell. Data are combined from 3 independent experiments. The mean is indicated. **(F)** Representative confocal images of HeLa cells with vacuolar and cytosolic *Salmonella* harboring pCHAR1-ProB.*mCherry* used for quantification in **(D,E)**. Digitonin permeabilized cells were identified by anti-Calnexin (blue) staining. Bacteria immunolabeling was done with anti-common structural antigen (CSA). Arrow indicates vacuolar bacteria. Scale bars, 10 or 2 μm.

Having established that the pCHAR-ProB.*mCherry* reporters respond appropriately to G6P, we next assessed the functionality and fidelity of these reporters in intracellular bacteria. For this we used the HeLa cell infection model within which the cytosolic and vacuolar populations of *Salmonella* have been characterized. Using the gentamicin protection assay we found that neither invasion nor net intracellular replication were affected in *Salmonella* harboring pCHAR1-ProB.*mCherry* (Figure 7A) or pCHAR2-ProB.*mCherry* (Figure 7B). Furthermore, by immunofluorescence microscopy we showed that pCHAR1-ProB.*mCherry* had no effect on the percentage of infected cells containing >50 bacteria per cell at 6 hpi, indicating that the plasmid has no effect on the ability of *Salmonella* to initiate hyper-replication in the cytosol (Figure 7C).

Given that pCHAR1-ProB.*mCherry* contains the truncated P*uhpT* and therefore produces less GFP under inducing conditions, one concern was that it might not accurately report cytosolic bacteria. We therefore utilized fluorescence microscopy to evaluate GFP fluorescence of intracellular bacteria in cells infected with bacteria bearing pCHAR1-ProB.*mCherry* at 6 hpi (Figures 7D–F). For mCherry and GFP controls, we included pCHAR1 (which lacks the ProB.*mCherry*-TT cassette) and the promoterless GFP control pMPMA3ΔPlac-*gfp* (Pnull.*gfp*). Rather than measure the fluorescence of individual bacteria, which are difficult to accurately resolve in cells containing high numbers, we used a region of interest (ROI) approach to select populations of cytosolic bacteria (C = >50 bacteria/cell) or vacuolar bacteria (V = ≤ 20 bacteria/cell). For bacteria bearing pCHAR1-ProB.*mCherry*, mCherry fluorescence was used to define the ROI, whereas for bacteria bearing the single reporter pCHAR1 or Pnull.*gfp* control plasmid, immunolabeling with an anti-CSA (common structural antigen of *Salmonella* LPS) antibody was used. GFP fluorescence (intensity/ROI pixel area) was high in cytosolic, but not vacuolar populations of bacteria bearing either of the CHAR reporters (Figures 7D,F). No significant difference was observed in fluorescence intensity between GFP-positive bacteria bearing pCHAR1-ProB*.mCherry* or pCHAR1 (0.61 ± 0.1 and 0.53 ± 0.14, mean ± *SD*, respectively). GFP fluorescence in vacuolar bacteria bearing pCHAR plasmids was no higher than that of bacteria harboring Pnull.*gfp*. To further verify the fidelity of the cytosolic GFP reporters, i.e., that GFP is only produced in cytosolic bacteria, we used digitonin permeabilization assay to specifically immunostain cytosolic bacteria (Knodler et al., 2010; Finn et al., 2017; Figure 7E). Together, these results conclusively show the bidirectional sensor, pCHAR1-ProB*.mCherry*, accurately identifies cytosolic bacteria without affecting the ability of *Salmonella* to establish either intracellular niche.

**Figure 8:**
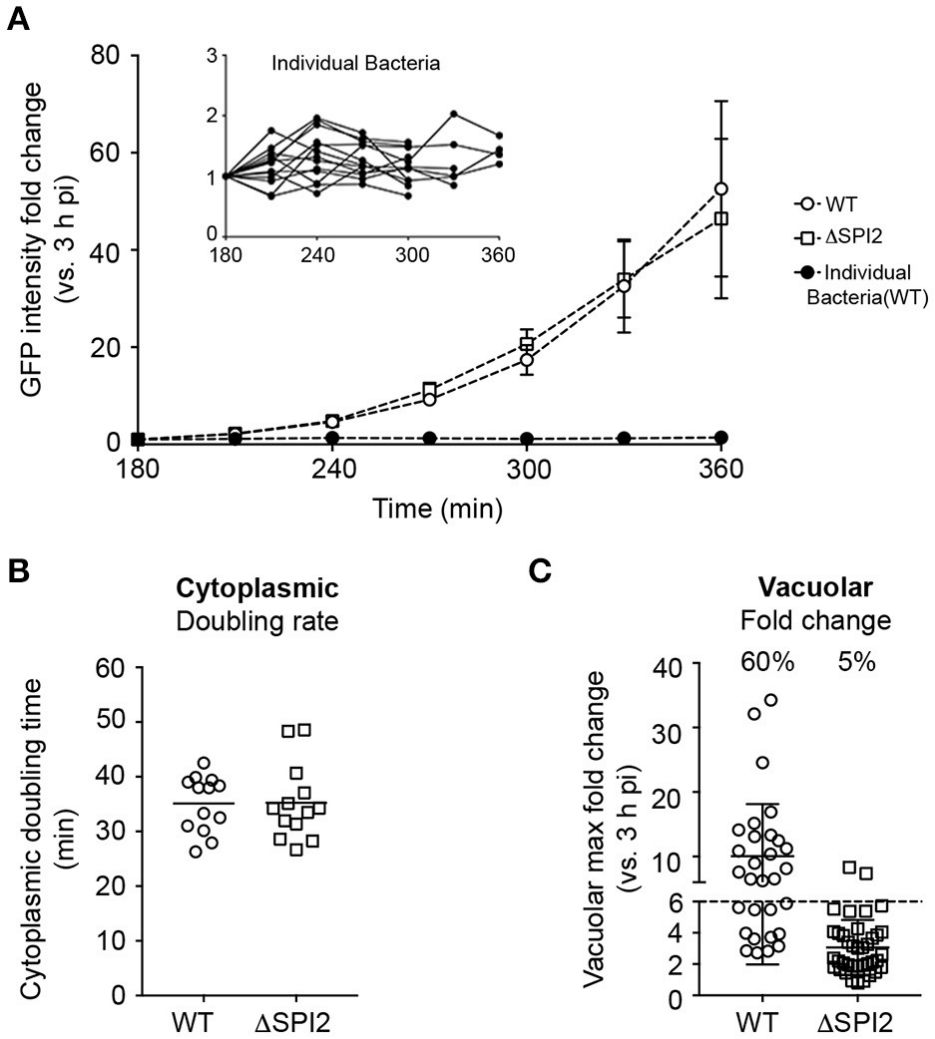
Use of pCHAR2-ProB.*mCherry* to compare replication rates in intracellular *Salmonella* by live cell imaging. Live cell imaging of HeLa cells infected with *Salmonella* harboring pCHAR2-ProB.*mCherry* between 3 and 22 hpi. **(A)** Fluorescent intensity of cells containing GFP+ cytosolic bacteria increases with time. Inset: The fluorescent intensity of individual GFP+ cytosolic bacteria does not increase with time. **(B)** The doubling time of cytosolic bacteria during log growth was calculated using GFP intensity over time. **(C)** The maximum fold change in mCherry intensity vs. 3 hpi was calculated to determine the vacuolar replication rate from the same image sets. The percentage of cells with a fold change >6 is reported. Each data point represents an infected cell. Data are combined from three independent experiments. The mean is indicated.

One interest in our lab is the heterogeneity of intracellular *Salmonella*, for example the different growth rates in vacuolar vs. cytosolic populations (Malik-Kale et al., 2012). Previously, we performed live cell imaging using *Salmonella* constitutively producing either mCherry or GFP together with a vacuolar content marker (fluorescent dextran), which was internalized by fluid phase uptake into the endocytic pathway prior to internalization of bacteria (Malik-Kale et al., 2012). While this approach did demonstrate that cytosolic bacteria (dextran –ve) replicate faster than those in the SCV (dextran +ve), lack of dextran signal around a bacterium does not definitively prove that they are cytosolic. An advantage of the pCHAR-ProB.*mCherry* constructs, or similar dual color reporters, is that the bacteria themselves report their intracellular localization; in this case, cytosolic (red and green) vs. vacuolar (red only). Since, for live cell imaging, it is generally advantageous to have a stronger fluorescent signal we selected for these experiments pCHAR2-ProB.*mCherry*, which contains the full length P*uhpT*. Infected HeLa cells were imaged using a spinning disc confocal system, as previously described (Malik-Kale et al., 2012). WT *Salmonella* were compared to a SPI2 deletion mutant (ΔSPI2), which has a replication defect in the vacuolar compartment but not in the cytosol (Malik-Kale et al., 2012). As shown in Figure 8A, the total GFP intensity per infected HeLa cell started increasing between 180 and 240 min pi, which is when cytosolic replication is initiated. Since the fluorescence intensity of individual bacteria did not change during this period (see inset in Figure 8A), the increase in fluorescence was due to replication of bacteria. Doubling times of bacteria in the cytosol were calculated by measuring the total integrated GFP fluorescence intensity of each infected cell over time (Figure 8B). No significant difference was detected between the two strains (doubling rate 35 min ± 5.2 for WT vs. 35 min ± 6.9 for ΔSPI2). A slightly different approach was used for vacuolar bacteria (red only), which replicate more slowly. The maximal fold increase of this population was calculated per cell by dividing the maximal intensity (X hpi) by the intensity at the starting point of imaging (3 hpi), yielding the fold increase. This showed that, as expected, in the vacuole the SPI2 mutant replicated less than the WT, 3 ± 1.8 vs. 10 ± 8 (mean ± *SD*), respectively (Figure 8C). These results validate that pCHAR2-ProB.*mCherry* is well suited for live cell imaging of *Salmonella*.

## Discussion

We have taken advantage of recent innovations in the field of synthetic biology, with the goal of improving episomal expression in *Salmonella*. Specifically, we incorporated synthetic insulated promoters as well as a synthetic transcriptional terminator into two expression plasmids, pFPV25.1 and pWSK29 (Valdivia and Falkow, 1996; Knodler et al., 2005). The resulting constructs enable predictable, tunable, constitutive expression of both heterologous and endogenous proteins and are a valuable addition to the study of host pathogen interactions, particularly at the single cell level.

Previously, we and others have found that plasmid-based expression of proteins can have deleterious effects on invasion and/or replication of intracellular *Salmonella* (Wendland and Bumann, 2002; Knodler et al., 2005; Helaine et al., 2010). While some of these effects may be avoided by chromosomal integration (Clark et al., 2009), it is often more efficient and practical to use plasmid based expression. Ideally, an expression vector should include a set of optimally configured genetic elements, of which two of the most important are the promoter, including a ribosomal binding site, and the transcriptional terminator (Hannig and Makrides, 1998). Often the major design goal is to maximize protein production, however when designing plasmids to be used in the study of pathogenesis, this must be balanced with the potential cost to the organism under a variety of environmental stresses. Here we replaced native components with synthetic biological parts that operate independently of either the genetic context or environment. The resultant series of pCON plasmids provide a straightforward approach to consistently and predictably produce proteins under a variety of conditions, while avoiding any negative impact on the host organism.

The use of synthetic promoters and transcriptional terminator in the pCON series of plasmids make protein production more predictable and consistent, however, as revealed by the comparison of GFP and mCherry production, insert-dependent variations are always possible. In addition to promoter activities, protein synthesis rates are also determined by other factors including; codon usage, mRNA stability, or protein maturation kinetics (Iizuka et al., 2011; Rosano and Ceccarelli, 2014). For any expression platform, these factors should be considered in the context of the ultimate goal. Here, our initial characterization of fluorescent protein production by the pCON plasmid series revealed that GFP fluorescence was growth phase independent whereas mCherry fluorescence fluctuated, possibly in response to different growth rates of the cells (Hebisch et al., 2013). Thus, in this system, GFP is more appropriate than mCherry for situations where protein production should depend linearly on promoter activity. However, mCherry is appropriate in situations where linearity is not required, as in when the readout is binary in nature. This was utilized in the development of the environmental sensors, pCHAR1-ProB.*mCherry* and pCHAR2-ProB.*mCherry*, which required only that red fluorescence was above a threshold in all bacteria whereas *gfp* expression must respond appropriately to the G6P signal. These biosensors provide a novel way to follow bacterial populations inside host cells, and should help to expand our knowledge of the roles of cytosolic vs. vacuolar bacteria in *Salmonella* pathogenesis. For example, they could be used for screening of *Salmonella* mutants in each of the subcellular niches, and thus facilitate the identification of genes affecting replication.

Being able to fine-tune production of proteins has far reaching applications. For example, there is intense interest in the use of *Salmonella* as a vaccine delivery platform. One hurdle in vaccine design is the optimization of antigen expression. High levels of antigen production must be balanced with the potential for metabolic costs, which can lead to decreased colonization and immune response in the host (Matic et al., 2009). Another potential use for the pCON series of plasmids is to generate consistently expressed sequences as reference standards for expression analysis by qRT-PCR or immunoblotting. There is currently no consensus on which standards to use; even the “housekeeping” genes, which are often used for reference, are not consistently and predictably expressed under all conditions. For example, *rpsM* which encodes the ribosomal protein S13 has been described as a housekeeping gene and used for constitutive expression in *E. coli* and *Salmonella* (Valdivia and Falkow, 1996; Malik-Kale et al., 2012; Nikolic et al., 2013), although it is subject to regulation by stress (Henard et al., 2014).

By building on the work of synthetic biologists, we have constructed a new generation of improved fluorescent protein expression vectors that allow fine-tuning of protein production levels. Additionally, we have demonstrated the applicability of this approach for the study of virulence factors, such as the T3SS effector, SopB. The ability to constitutively express T3SS effectors can complement current approaches to investigate protein function and effects on host cells. These constructs are a useful addition to the genetic tools available for the study of *Salmonella* biology, and can likely be extended to other closely related pathogens.

## Author contributions

OS-M, KC, AC, TS, CF conceived and designed the experiments. KC, AC, TS, CF performed experiments. KC, AC, TS, CF analyzed data. OS-M, KC wrote the manuscript.

### Conflict of interest statement

The authors declare that the research was conducted in the absence of any commercial or financial relationships that could be construed as a potential conflict of interest.

## Abbreviations

GFP: Green Fluorescent Protein
pi: post-infection
SCV: *Salmonella*-containing vacuole
synTT: synthetic transcriptional terminator
MFI: mean fluorescence intensity
SPI: *Salmonella* Pathogenicity Island
T3SS: type three secretion system
G6P: glucose-6-phosphate
ROI: region of interest
CHAR: cytosolic hexose phosphate activated reporter.
